# Supplementary figures and images for: Cytohesin-2 is essential for the perinatal development of mice and regulates Golgi volume
Source: Life Sci Alliance. 2026 Feb 11;9(5):e202503429. doi: 10.26508/lsa.202503429 (PMC12894763; doi:10.26508/lsa.202503429)

SourceDataForFigureS1A

Original blots used for Fig S1A and corresponding Ponceau stain

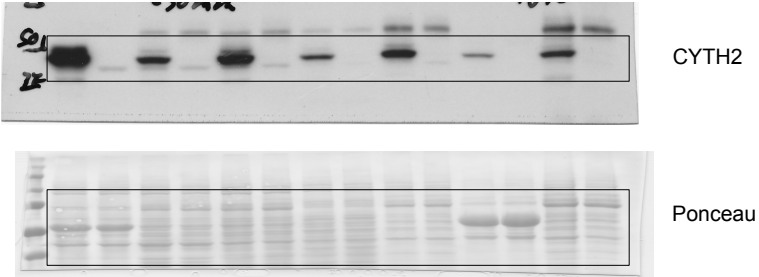

Supplement: Supplementary file 1 [file LSA-2025-03429_SdataFS1.pdf]
