# Supplementary material for: Cytohesin-2 is essential for the perinatal development of mice and regulates Golgi volume
Source: Life Sci Alliance. 2026 Feb 11;9(5):e202503429. doi: 10.26508/lsa.202503429 (PMC12894763; doi:10.26508/lsa.202503429)

SourceDataForFigureS4A

Original blots used for Fig S4A (glucose restimulation) and corresponding Ponceau stain

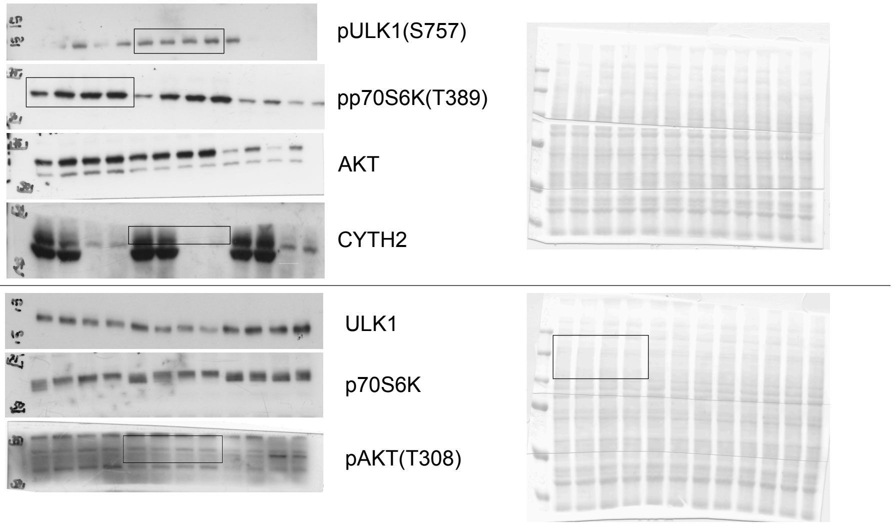

Phospho-ULK1(S757) and ULK1 normalized to Ponceau used for Fig S4A

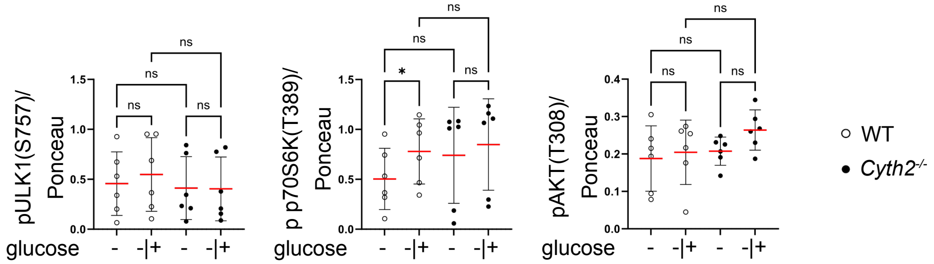

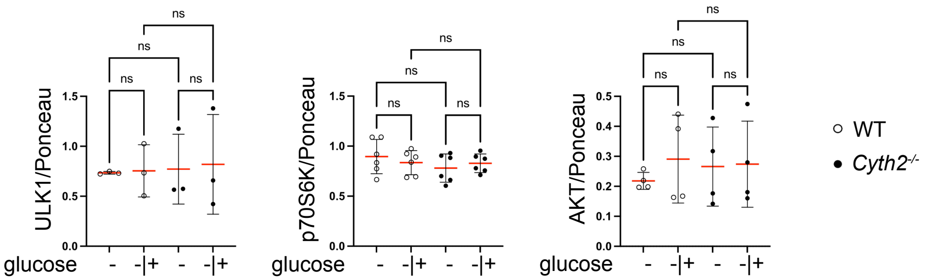

Supplement: Supplementary file 2 [file LSA-2025-03429_SdataFS4.1.pdf]
