# Supplementary material for: Cytohesin-2 is essential for the perinatal development of mice and regulates Golgi volume
Source: Life Sci Alliance. 2026 Feb 11;9(5):e202503429. doi: 10.26508/lsa.202503429 (PMC12894763; doi:10.26508/lsa.202503429)

SourceDataForFigureS4C

Original blots used for Fig S4C (amino acid restimulation) and corresponding Ponceau stain

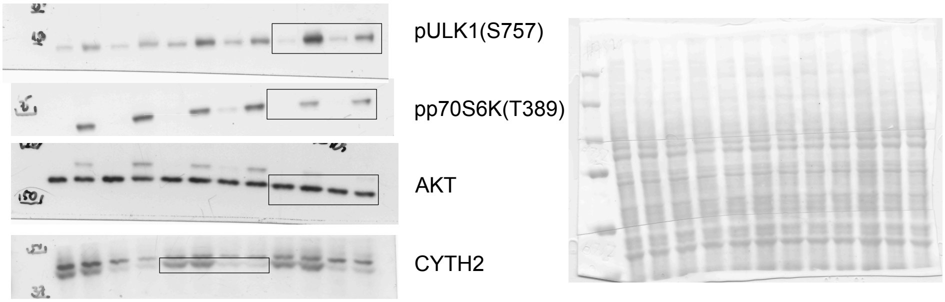

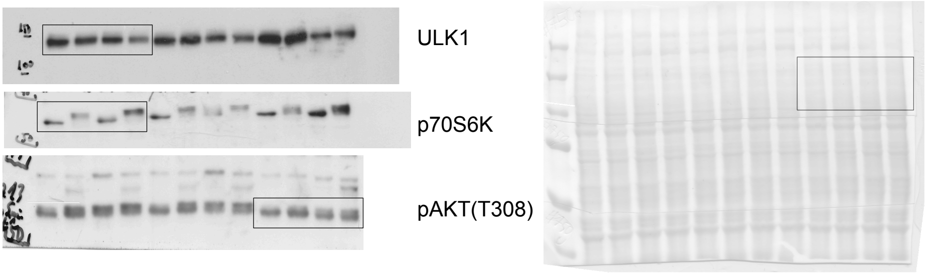

Phospho-ULK1(S757) and ULK1 normalized to Ponceau used for Fig S4C
Of note: Due to a clear batch effect of two membranes for six experiments detecting ULK1, values were normalized to the average intensity per membrane.

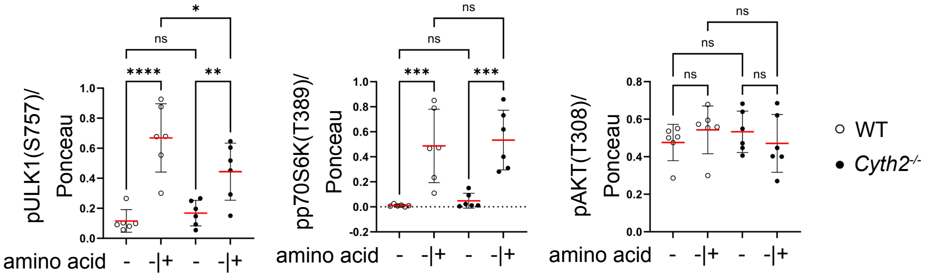

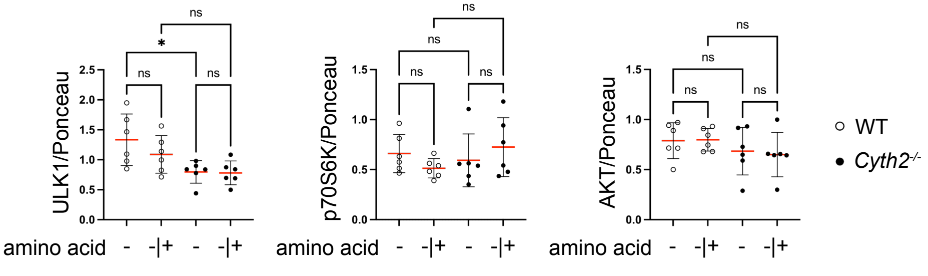

Supplement: Supplementary file 4 [file LSA-2025-03429_SdataFS4.3.pdf]
