# Supplementary material for: Cytohesin-2 is essential for the perinatal development of mice and regulates Golgi volume
Source: Life Sci Alliance. 2026 Feb 11;9(5):e202503429. doi: 10.26508/lsa.202503429 (PMC12894763; doi:10.26508/lsa.202503429)
Supplement: Supplementary file 6 [file LSA-2025-03429_SdataF4.pdf]

SourceDataForFigure4&6

Original blots used for Fig 4C-D and corresponding Ponceau stain

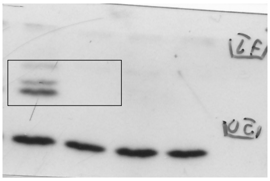

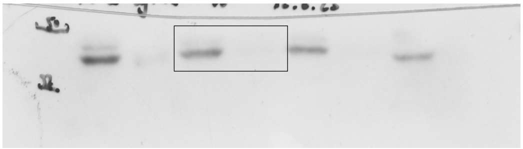

CYTH2

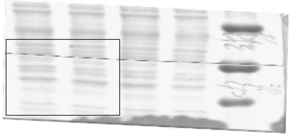

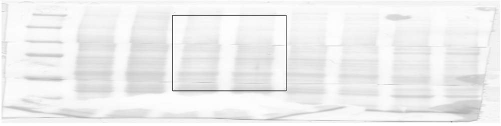

Ponceau

Original blots used for Fig 6F and corresponding Ponceau stain

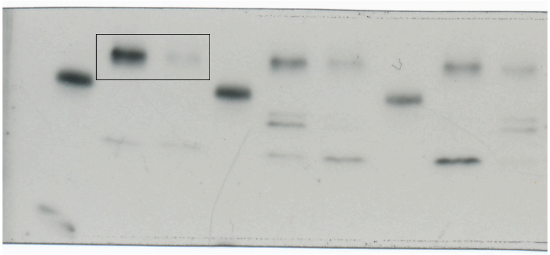

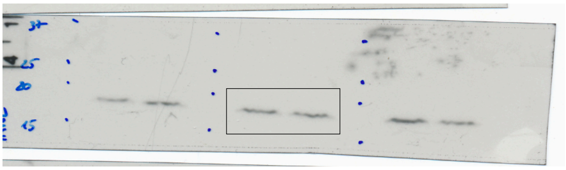

WFDC2

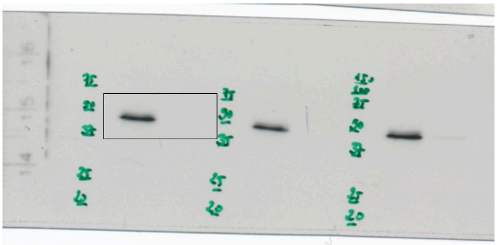

CYTH2

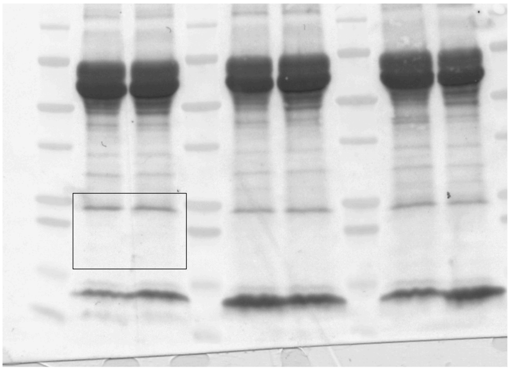

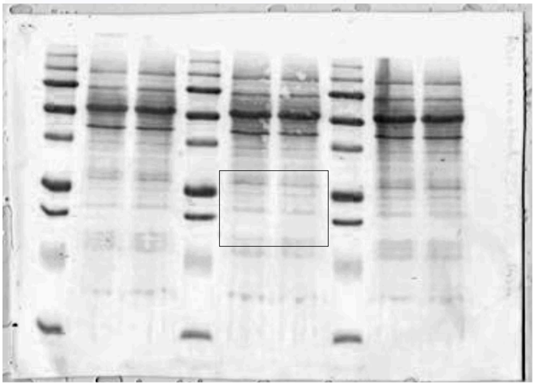

Ponceau
